# Antibodies to the Glycoprotein GP2 Subunit Cross-React between Old and New World Arenaviruses

**DOI:** 10.1128/mSphere.00189-18

**Published:** 2018-05-02

**Authors:** Fatima Amanat, James Duehr, Lisa Oestereich, Kathryn M. Hastie, Erica Ollmann Saphire, Florian Krammer

**Affiliations:** a Department of Microbiology, Icahn School of Medicine at Mount Sinai, New York, New York, USA; b Graduate School of Biological Sciences, Icahn School of Medicine at Mount Sinai, New York, New York, USA; c Department of Virology, Bernhard Nocht Institute for Tropical Medicine, Hamburg, Germany; d German Centre for Infection Research (DZIF), Partner Site Hamburg, Hamburg, Germany; e Department of Immunology and Microbiology, The Scripps Research Institute, La Jolla, California, USA; f Skaggs Institute for Chemical Biology, The Scripps Research Institute, La Jolla, California, USA; University of Maryland, College Park

**Keywords:** GP2, GPC, Junin, Lassa, Machupo, arenaviruses

## Abstract

Arenaviruses pose a major public health threat and cause numerous infections in humans each year. Although most viruses belonging to this family do not cause disease in humans, some arenaviruses, such as Lassa virus and Machupo virus, are the etiological agents of lethal hemorrhagic fevers. The absence of a currently licensed vaccine and the highly pathogenic nature of these viruses both make the necessity of developing viable vaccines and therapeutics all the more urgent. Arenaviruses have a single glycoprotein on the surface of virions, the glycoprotein complex (GPC), and this protein can be used as a target for vaccine development. Here, we describe immunization strategies to generate monoclonal antibodies (MAbs) that cross-react between the glycoprotein complexes of both Old World and New World arenaviruses. Several monoclonal antibodies isolated from immunized mice were highly cross-reactive, binding a range of Old World arenavirus glycoproteins, including that of Lassa virus. One such monoclonal antibody, KL-AV-2A1, bound to GPCs of both New World and Old World viruses, including Lassa and Machupo viruses. These cross-reactive antibodies bound to epitopes present on the glycoprotein 2 subunit of the glycoprotein complex, which is relatively conserved among arenaviruses. Monoclonal antibodies binding to these epitopes, however, did not inhibit viral entry as they failed to neutralize a replication-competent vesicular stomatitis virus pseudotyped with the Lassa virus glycoprotein complex *in vitro*. In addition, no protection from virus challenge was observed in *in vivo* mouse models. Even so, these monoclonal antibodies might still prove to be useful in the development of clinical and diagnostic assays.

**IMPORTANCE** Several viruses in the *Arenaviridae* family infect humans and cause severe hemorrhagic fevers which lead to high case fatality rates. Due to their pathogenicity and geographic tropisms, these viruses remain very understudied. As a result, an effective vaccine or therapy is urgently needed. Here, we describe efforts to produce cross-reactive monoclonal antibodies that bind to both New and Old World arenaviruses. All of our MAbs seem to be nonneutralizing and nonprotective and target subunit 2 of the glycoprotein. Due to the lack of reagents such as recombinant glycoproteins and antibodies for rapid detection assays, our MAbs could be beneficial as analytic and diagnostic tools.

## Abstract (precis)

Several viruses in the *Arenaviridae* family infect humans and cause severe hemorrhagic fevers which lead to high case fatality rates. Due to their pathogenicity and geographic tropisms, these viruses remain very understudied. As a result, an effective vaccine or therapy is urgently needed. Here, we describe efforts to produce cross-reactive monoclonal antibodies that bind to both New and Old World arenaviruses. All of our MAbs seem to be nonneutralizing and nonprotective and target subunit 2 of the glycoprotein. Due to the lack of reagents such as recombinant glycoproteins and antibodies for rapid detection assays, our MAbs could be beneficial as analytic and diagnostic tools.

## INTRODUCTION

Arenaviruses are a family of negative-sense RNA viruses commonly found in rodents (genus *Mammarenavirus*) and snakes (genus *Reptarenavirus*), but members of the *Mammarenavirus* genus are known to infect humans via contact or exposure to urine or feces of the reservoir host (1). *Mammarenavirus* viruses are divided into Old World (OW) and New World (NW) classifications based on three factors: phylogeny, serology, and geography (1). The genus consists of over 20 different viruses which are all found in distinct locations based on the availability of the natural rodent host. Lymphocytic choriomeningitis virus (LCMV) is an exception and is found throughout the world due to the wide presence of its rodent host, the house mouse (Mus musculus) (2). Although the *Arenaviridae* family consists of more than 25 species, only a few arenaviruses are known to infect humans and cause disease. One of the most prominent arenaviruses, Lassa virus (LASV), is endemic to West Africa and is known to induce a severe hemorrhagic fever and multiorgan system failure, often resulting in death (3). Another prominent example is Machupo virus (MACV), an NW arenavirus, known to cause Bolivian hemorrhagic fever in humans with a case fatality rate of 25 to 35% (4). The bisegmented RNA genome of these viruses encodes a total of four proteins including the polymerase (L), the matrix protein (Z), the nucleoprotein (NP), and the glycoprotein complex (GPC). The GPC is the only glycoprotein present on the surface and is cleaved into glycoprotein subunit 1 (GP1) and glycoprotein subunit 2 (GP2) by the protease SKI-1/S1P (5). Cleavage of the GPC is essential for infectivity; cells devoid of SKI-1/S1P are incapable of releasing infectious virions (6). In the past few years, it has been shown that inhibitors of SKI-1/S1P can be used as antiviral drugs against OW and NW arenaviruses (7, 8). Since the GPC is a surface-exposed component of functional virions essential for engagement and entry into host cells via alpha-dystroglycan (OW) (9) or transferrin receptor 1 (NW) (10), the GPC is an obvious target for vaccine and/or therapeutic development. Here, using two different immunization strategies in mice, we aimed at inducing and isolating antibodies that are cross-reactive between phylogenetically distant arenavirus GPCs. We have generated a panel of six mouse monoclonal antibodies (MAbs) to the GPC. All six MAbs target GP2 and show broad cross-reactivity between OW arenaviruses, with one MAb binding broadly to both OW and NW arenaviruses.

## RESULTS

### Generation of recombinant proteins.

**FIG 1:**
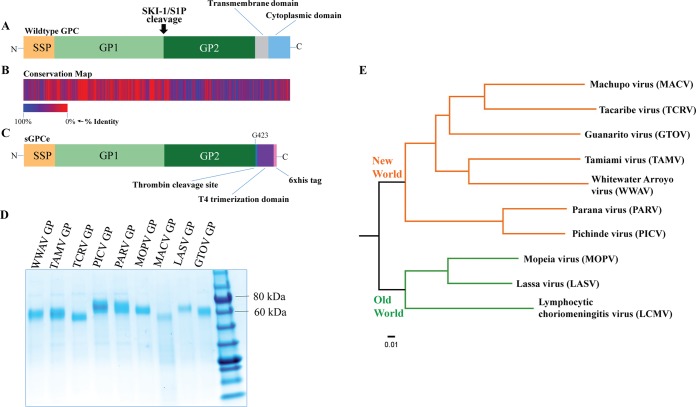
Recombinant glycoproteins from both New World and Old World arenaviruses were cloned and analyzed to determine amino acid similarity. (A) Schematic of wild-type arenavirus GPC. The full-length GPC of arenaviruses is depicted including the stable signal peptide (SSP), glycoprotein 1 subunit (GP1), glycoprotein 2 subunit (GP2), transmembrane domain, and cytoplasmic domain. The site of cleavage by SKI-1/S1P is also indicated. (B) Amino acid conservation map of GPCs based on the nine divergent arenavirus strains used to generate our recombinant constructs plus the prototypic arenavirus LCMV. The amino acid sequences of all 10 GPCs were aligned in a multiple sequence alignment using Clustal Omega and then evaluated for conservation using AACon via a Jalview applet. The conservation values were then exported and visualized in Microsoft Excel. The map is based on amino acid identity. (C) Schematic of recombinant GPC constructs. Residues 1 to 423 coding for the ectodomain of the GPCs were fused to the T4 fibritin trimerization domain and cloned into a shuttle vector used to express recombinant protein in the baculovirus expression system. (D) SDS-PAGE of the nickel-nitrilotriacetic acid-purified recombinant GPCs. All nine recombinant GPCs were analyzed on an SDS-PAGE gel under denaturing conditions to assess size and purity. The GPCs were found to be approximately 65 to 70 kDa, and differences in size are likely caused by different glycosylation patterns. (E) Phylogenetic analysis of the nine GPCs expressed. The amino acid sequences were aligned and compared, and a tree was constructed to visualize relative distance. The scale bar represents a 1% change in amino acids.

To produce recombinant soluble GPCs derived from arenaviruses for vaccination and antibody characterization, we utilized a conserved glycine upstream of the transmembrane domain as a termination point for expression of the ectodomain alone. We also fused it to a C-terminal T4 trimerization domain to enhance stability and a hexahistidine tag to facilitate purification (11). These modifications allowed for efficient and productive expression of a construct based on the ectodomain of the GPC, termed sGPCe (Fig. 1A and C). Conservation analysis was also performed to create a map based on amino acid identity that pinpoints regions with high and low levels of amino acid identity. This analysis shows that amino acids differ highly for the GP1 subunit while amino acids in GP2 and the stable signal peptide (SSP) are more conserved among the 10 glycoproteins chosen (Fig. 1B). This also suggests that epitopes that are conserved between different arenaviruses might exist on both the SSP and the GP2 subunits. We then selected two OW arenaviruses, Lassa virus (LASV) and Mopeia virus (MOPV), as well as seven NW arenaviruses, Machupo virus (MACV), Tacaribe virus (TCRV), Guanarito virus (GTOV), Tamiami virus (TAMV), Whitewater Arroyo virus (WWAV), Parana virus (PARV), and Pichinde virus (PICV), for recombinant protein expression via the baculovirus system. All purified glycoproteins showed a single band in the range of 60 to 70 kDa on a reducing sodium dodecyl sulfate-polyacrylamide gel electrophoresis (SDS-PAGE) gel, respectively (Fig. 1D). The differences in size observed are likely caused by the different glycosylation patterns of each glycoprotein since the theoretical mass of all expressed proteins is between 52 and 56 kDa. In order to get a better understanding of the phylogenetic relationships between these arenavirus GPCs, the amino acid sequences of representative arenavirus glycoproteins, with the addition of LCMV GPC, were aligned, and a phylogenetic tree was constructed to visualize relative distances (Fig. 1E). Notably, the GPCs of Old World viruses such as Lassa virus are highly divergent from the GPCs of New World viruses such as MACV or GTOV.

### Generation of MAbs via murine hybridoma technology.

We used two different strategies to induce anti-GPC antibodies. The first strategy was geared toward inducing LASV-specific antibodies. For this, mice were vaccinated with DNA vaccines encoding the LASV GPC (strain Nig08-A19) three times followed by a protein boost using recombinant LASV GPC of the same strain. This strategy yielded three MAbs, KL-AV-1A2, KL-AV-1A12, and KL-AV-1B3. The second approach was based on a strategy that we successfully developed for the generation of cross-reactive anti-influenza virus hemagglutinin and neuraminidase antibodies (12–15). Here, we sequentially vaccinated mice with DNA vaccines encoding the GPC of LASV followed by the GPCs of MOPV and MACV with a final LASV GPC recombinant protein boost. The goal of this sequential vaccination was to refocus the antibody response toward conserved epitopes shared by the GPCs of all three divergent arenavirus species. Although MOPV and LASV are both OW arenaviruses, the GPC sequences can differ to a great extent. Another reason to use these two GPCs was to explore whether epitopes exist that are conserved among one OW and one NW virus but not all OW viruses. This strategy also resulted in three MAbs, KL-AV-1G12, KL-AV-1H9, and KL-AV-2A1. The six MAbs and their characteristics are described in Table 1.

**TABLE 1:** Name, vaccination regimen used, and isotype of the six MAbs

Name	Regimen	Isotype
KL-AV-1A2	LASV GPC, 3 times	IgG2a
KL-AV-1A12	LASV GPC, 3 times	IgG2a
KL-AV-1B2	LASV GPC, 3 times	IgG2a
KL-AV-1G12	Sequential LASV GPC-MACV GPC-MOPV GPC	IgG2b
KL-AV-1H9	Sequential LASV GPC-MACV GPC-MOPV GPC	IgG2a
KL-AV-2A1	Sequential LASV GPC-MACV GPC-MOPV GPC	IgG2a

### The isolated MAbs show broad binding to recombinant arenavirus GPC proteins and recombinant vaccinia viruses pseudotyped with arenavirus GPCs.

**FIG 2:**
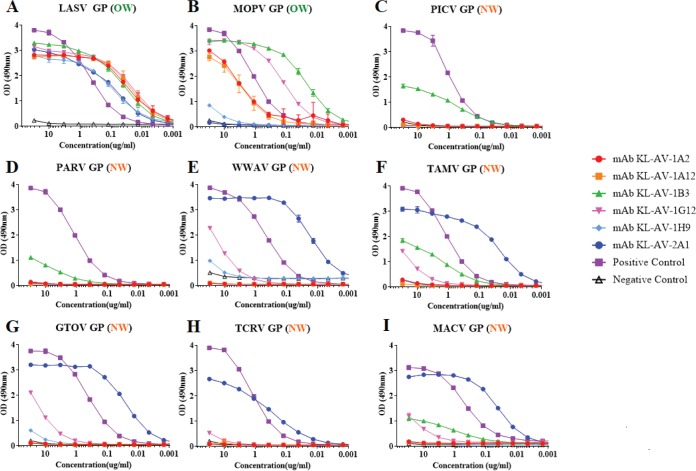
The generated murine MAbs are highly cross-reactive and bind divergent arenavirus GPCs. (A to I) ELISA against all recombinant GPCs. A standard ELISA was performed using the nine different GPCs to test binding of the six monoclonal antibodies. Plates were coated with 2 µg/ml of each GPC, and 30 µg/ml of each MAb was used as the starting point followed by 1:3 dilutions. Naive mouse serum was used as a negative control, while an antihistidine antibody was used as a positive control.

**FIG 3:**
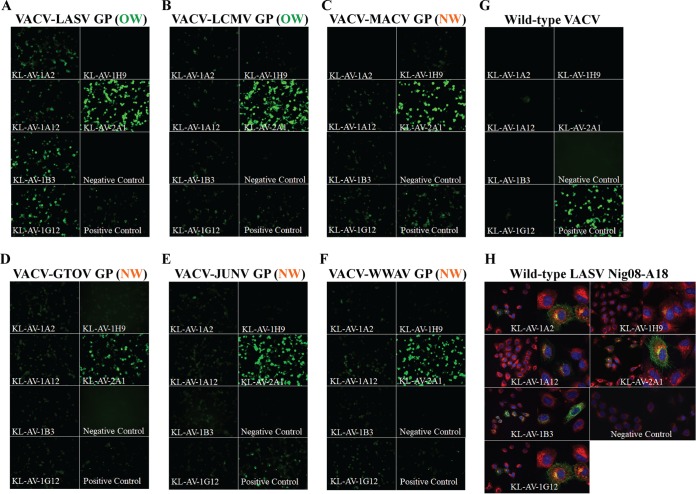
The isolated MAbs bind to cells infected with recombinant vaccinia viruses expressing various arenavirus GPCs as well as to cells infected with authentic LASV. (A to F) Immunofluorescence images of nonpermeabilized BSC40 cells infected with vaccinia viruses expressing different arenavirus GPCs. Cells were infected at an MOI of 1 overnight with different recombinant vaccinia viruses that express arenavirus GPCs and stained with the six MAbs at 30 µg/ml. Naive mouse serum was used as a negative control, while polyclonal serum from a mouse vaccinated with LASV GPC, MOPV GPC, and MACV GPC was used as a positive control at a dilution of 1:100. (G) Immunofluorescence images of cells infected with wild-type VACV and stained with the six antibodies. Cells were infected at an MOI of 1 overnight with wild-type VACV and stained with the six antibodies. Serum from a mouse vaccinated with MVA was used as a positive control. (H) Immunofluorescence images of Vero.E6 cells infected with LASV strain Nig08-A18 and stained with the six different MAbs. Vero.E6 cells were infected with wild-type LASV in a BSL-4 facility for 24 h at an MOI of 0.1. Cells were fixed, permeabilized, and stained with 10 ng/µl of each MAb. Cell masks and cell nuclei (DAPI) were also stained, and the merged images are shown.

After hybridoma generation and initial screens, purified antibodies were tested in an enzyme-linked immunosorbent assay (ELISA) for reactivity against nine different arenavirus GPCs (Fig. 2A to I). All six of the isolated MAbs bound well to LASV sGPCe (Fig. 2A). Interestingly, the binding pattern observed for the LASV sGPCe differs from that of the closely related MOPV sGPCe, for which all MAbs tested (excluding KL-AV-1B3 and KL-AV-1G12) lost some of their binding activity (Fig. 2B). One MAb, KL-AV-2A1, showed strong binding activity to glycoproteins derived from WWAV, TAMV, GTOV, TCRV, and MACV but not MOPV, PICV, and PARV. As a result of this cross-binding to OW and NW viruses, KL-AV-2A1 can be considered a broadly cross-reactive antiarenavirus GPC antibody. Another MAb, KL-AV-1B3, also showed broad binding activity, but at a very low level (Fig. 2). Since the glycoproteins used for the ELISAs were recombinant in nature and their conformation is as yet unknown, it was essential to assess whether the MAbs tested can also bind to the glycoprotein in its native conformation on the surface of an infected cell. Hence, BSC40 cells were infected at a multiplicity of infection (MOI) of 1.0 with various recombinant vaccinia viruses (VACV) that expressed glycoproteins derived from arenaviruses (16). Immunofluorescence (IF) staining with the MAbs was performed on cells infected with VACV-LASV GPC, VACV-LCMV GPC, VACV-MACV GPC, VACV-GTOV GPC, VACV-JUNV GPC (expressing the GPC of the NW arenavirus Junin virus [JUNV]), and VACV-WWAV GPC (Fig. 3). The staining patterns observed confirmed binding of all MAbs to the GPC of LCMV, another OW arenavirus. This is consistent with results observed via ELISA in which five out of six MAbs bound to both OW virus GPCs (LASV and MOPV) tested, albeit at different levels. Furthermore, reactivity of KL-AV-2A1 to JUNV GPC (not tested in ELISA [Fig. 3E]) was observed. In general, findings from the IF staining were consistent with the ELISA results. The exception is KL-AV-1H9, which exhibited no binding to infected cells but had good reactivity via ELISA. In order to be certain that the antibodies were binding specifically to the GPCs of arenaviruses, cells were also infected with wild-type VACV and then stained with the six antibodies (Fig. 3G). None of the antibodies stained these infected cells. Serum from a mouse which was vaccinated with modified vaccinia virus Ankara (MVA) was used as a positive control, and this serum bound well to cells infected with wild-type VACV.

### Authentic GPC is recognized on infected cells.

All six monoclonal antibodies were further tested for binding to the glycoprotein on the authentic LASV strain Nig08-A18 in a biosafety level 4 (BSL-4) facility. Vero.E6 cells were infected with the authentic virus at an MOI of 0.1 for 24 h and then fixed and permeabilized. All MAbs bound to infected cells with the exception of KL-AV-1H9, which also did not bind cells infected with the recombinant vaccinia viruses (Fig. 3H) but did bind in ELISA to recombinant GPCs. It is possible that the antibody binds an epitope exposed in the recombinant glycoprotein but not on the authentic glycoprotein, as it is presented on the surface of an infected cell.

### Broadly reactive MAbs target linear or microconformational epitopes on the conserved GP2 subunit.

**FIG 4:**
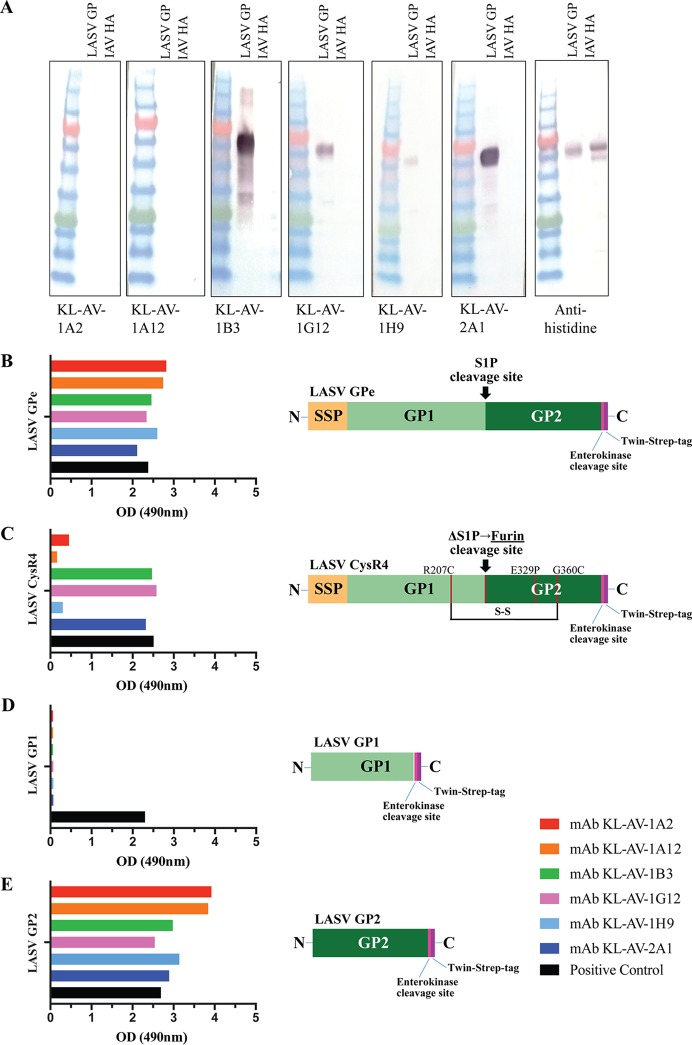
Some MAbs bind to linear epitopes, while all MAbs bind to the GP2 subunit of the LASV glycoprotein. (A) Western blot analysis. Recombinant LASV GP was denatured, and a Western blot assay was performed to see whether antibodies bind to linear or conformational epitopes. The blot was probed with the six MAbs at 30 µg/ml, and an anti-mouse–alkaline phosphatase (AP) antibody was used as a secondary stain. An AP conjugate kit was used to visualize the blot. To ensure specificity of the MAbs to LASV GPC, an irrelevant protein (influenza virus hemagglutinin [IAV HA]) was run on each blot. (B to D) Subunit mapping analysis for each MAb. Reactivity is shown for LASV GPe (B), prefusion stabilized LASV GPCysR4 (30) (C), the LASV GP1 subunit alone (D), and the LASV GP2 subunit alone (E). A schematic for each construct is displayed to the right of each ELISA graph. The human antibody 22.5D, which recognizes a linear epitope on GP2, was used as a positive control for panels B, C, and E, while the GP1-specific antibody 3.3B was used as a positive control for panel D (28). All MAbs were used at a concentration of 5 µg/ml. OD, optical density.

To test whether the monoclonal antibodies bind to linear or conformational epitopes, a Western blot analysis was performed with LASV sGPCe under reducing and denaturing conditions. The membrane was probed with 30 µg/ml of each of the six monoclonal antibodies, and reactivity of MAbs KL-AV-1B3, KL-AV-1G12, KL-AV-1H9, and KL-AV-2A1 was observed (Fig. 4A), suggesting that a linear epitope is targeted by these antibodies. To map the epitopes of the MAbs, we tested all the MAbs in ELISA using four different recombinant GPC constructs. All MAbs bound to LASV GPe, which encodes residues 1 to 424 of LASV GPC (Fig. 4B). Next, the MAbs were tested for binding to another recombinant construct, LASV GPCysR4, which also encodes residues 1 to 424 but is engineered to maintain the prefusion conformation. KL-AV-1B3, KL-AV-1G12, and KL-AV-2A1 bound very well to this modified GPC, but the other three MAbs had little to no binding (Fig. 4C). The antibodies were further tested for binding to another construct, LASV GP1, which encodes GP1 residues 74 to 237. None of the MAbs bound to the recombinant LASV GP1 subunit (Fig. 4D). Last, another ELISA was performed using a recombinant LASV GP2 subunit (residues 260 to 424) as the substrate, and all of the six MAbs bound well to this construct (Fig. 4E). Binding to both the full-length LASV GPe and the GP2 subunit alone indicates that all the antibodies target an epitope present on the GP2 subunit.

### The isolated GP2 binding MAbs show negligible functionality *in vitro* and *in vivo.*

**FIG 5:**
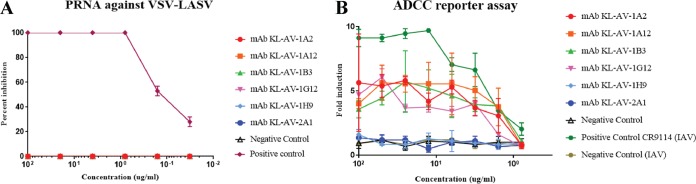
The six MAbs do not neutralize VSV-LASV and show low ADCC reporter assay activity. (A) PRNA against VSV-LASV. Plaque reduction neutralization assays were performed using a VSV expressing the LASV GPC instead of its own G protein. Each MAb was incubated with VSV-LASV for 1 h, and this mixture was then used to infect Vero.E6 cells for 1 h. The mixture was then removed, and the cells were grown for 48 h with antibody also present in the agar overlay to see if the plaque number or size was reduced. An anti-influenza virus antibody was used as a negative control, while a human antibody, 25.10C, was used as a positive control. (B) ADCC reporter activity of MAbs using VSV-LASV-infected Vero.E6 cells as the substrate. All MAbs were tested in a simple ADCC reporter assay in which Vero.E6 cells were infected overnight with VSV-LASV and the MAbs and effector cells were added onto the infected cells the next morning. Luminescence was measured at the end of the assay to assess ADCC activity. The human anti-influenza virus hemagglutinin stalk MAb CR9114 was used on H4N6 (influenza A virus [IAV])-infected Vero.E6 cells as a positive control.

To test if the isolated MAbs have *in vitro* neutralization activity, plaque reduction neutralization assays (PRNAs) were performed using a recombinant vesicular stomatitis virus that expresses the LASV GPC (VSV-LASV). Since this virus relies completely on the LASV GPC for entry due to the lack of its own G protein, any inhibition of entry of this virus would indicate specific anti-LASV GPC neutralization (17–19). We observed that none of the six antibodies led to a reduction in plaque numbers, and thus, the antibodies do not neutralize VSV-LASV (Fig. 5A). To ensure that the assay was functional, a known human antibody which neutralizes LASV, 25.10C, was used as a positive control (20).

**FIG 6:**
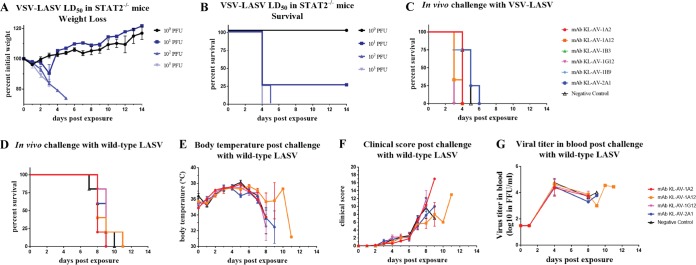
The isolated MAbs are not protective in two different mouse challenge models. (A) mLD_50_ of VSV-LASV. *Stat2*^*−/−*^ mice were infected with different doses of VSV-LASV via the intraperitoneal route to calculate the LD_50_, and mice were weighed for 14 days after challenge with the virus. (B) Survival curve of mice infected with VSV-LASV. The survival curve of the LD_50_ experiment is shown for the four groups, each of which received a different dose of virus as indicated. (C) Protective efficacy of antibodies against VSV-LASV. All six antibodies were tested for their ability to protect against infection in a prophylactic setting in which antibodies were injected into mice 2 h prior to infection with VSV-LASV. (D) Protective efficacy of MAbs against authentic LASV. The protective efficacy of four of the antibodies was tested against authentic LASV (Nig08-A18 strain). Antibodies were injected into mice 2 h prior to infection, and survival was monitored for 14 days. (E) Body temperature post-challenge with authentic LASV. The body temperature of all mice was recorded daily after viral challenge to observe development of a fever. (F) Clinical score of each group post-challenge with authentic LASV. Symptoms were monitored and clinical score was recorded daily for each group after challenge to assess establishment of disease caused by the virus. (G) Viral titers in the blood post-challenge with authentic LASV. To analyze the amount of virus in the blood of mice receiving the four different MAbs, blood samples were taken at different time points and an immunofocus assay was performed to quantify the amount of virus present.

Recent reports have shown that neutralization is not an absolute requirement for a MAb to protect *in vivo* against influenza and Ebola viruses (21–25). Specifically, murine IgG2a and IgG2b MAbs have exhibited protection in the mouse model driven by effector functions. To investigate this possibility, we performed a simple reporter assay (antibody-dependent cellular cytotoxicity [ADCC] reporter assay) that measures interactions between murine mFcγRIV (most abundant on murine natural killer cells) and the Fc regions of antibodies bound to VSV-LASV-infected cells. Fc-FcR engagement in this assay leads to the expression of luciferase, which can then be quantified (23). Four out of six MAbs elicited a signal at relatively high MAb concentrations (Fig. 5B), but the signal appeared weak compared to a positive-control MAb (human CR9114 against cells infected with influenza A virus). Nevertheless, the small effect observed in the ADCC reporter assay could potentially lead to protection *in vivo*. To test this, for cost and ease of access concerns, we first established a mouse model for *in vivo* protection studies that can be handled outside a BSL-4 laboratory. In the past, we have employed *Stat2*^*−/−*^ mice to study the pathogenicity of viruses that do not exhibit a high degree of pathogenicity in wild-type mice. In the *Stat2*^*−/−*^ background, we have observed that a VSV expressing the Ebola virus glycoprotein showed a high level of pathogenicity which could be protected against by using neutralizing and ADCC-active MAbs (while being completely attenuated in *Stat2*-competent mice [16]). We therefore infected *Stat2*^*−/−*^ mice with increasing doses of VSV-LASV. Indeed, this vaccine strain also induced pathogenicity in the knockout mice, and a 50% murine lethal dose (mLD_50_) of 4.6 PFU was established (Fig. 6A and B). We then tested the ability of the six MAbs to protect these mice from infection in a prophylactic setting where 10 mg of each antibody/kg of body weight was injected into mice, via the intraperitoneal route, 2 h before infection. Mice were then infected with 10 mLD_50_s of VSV-LASV. None of the six antibodies tested provided a survival benefit to the mice compared to a control MAb, and all mice succumbed to infection (Fig. 6C).

In parallel, a similar experiment was conducted with a subset of the MAbs using wild-type LASV, strain Nig08-A18, in chimeric mice that are susceptible to wild-type LASV. In this experiment, mice were treated prophylactically with 15 mg/kg of MAbs KL-AV-1A2, KL-AV-1A12, KL-AV-1G12, and KL-AV-2A1 and then challenged with the authentic Lassa virus in a BSL-4 setting (Fig. 6D). Only these four MAbs were tested due to the availability of a limited number of mice. Body temperatures were also recorded daily after viral challenge (Fig. 6E). However, no significant difference among the five groups was observed. Clinical symptoms were monitored daily, and scores were assigned for consciousness, appearance, eyes, and respiration (Fig. 6F). Additional points were given for diarrhea, weight loss, and body temperature (details in Materials and Methods). While a trend toward a small but not significant benefit in terms of clinical signs was noticeable for one of the MAbs (KL-AV-1A12), the overall results mirrored the results observed in the *Stat2*^*−/−*^ mouse model in terms of survival. Finally, to see if the amounts of virus in the blood of the mice in the five groups differed, blood samples from different time points were titrated (Fig. 6G). There did not seem to be any significant difference in viral titers in the blood between the groups.

## DISCUSSION

Currently, no vaccines or antivirals are available to effectively treat OW arenaviruses. The same is true for most NW arenaviruses, except for a specific vaccine for JUNV (Candid #1) that is being used in Argentina (26). Ribavirin has occasionally been used off-label for treatment, transfer of convalescent-phase plasma is an established treatment option for JUNV infections, and a JUNV MAb therapeutic is in early stages of development (27–29). Even so, there remains a need to better understand the antigenicity of the arenavirus GPC to aid the development of future vaccines and therapeutics. Here, we used two different vaccination strategies to induce antibodies against the GPC of arenaviruses. The first strategy used was geared toward inducing LASV-specific MAbs, and only the LASV GPC was used for sequential vaccination. This strategy led to two MAbs specific for OW virus GPC with strong binding to LASV and weaker binding to MOPV and one MAb that bound very well to both LASV and MOPV and had some residual binding to some NW virus GPCs. The second scheme was based on sequential vaccination (30) of animals with GPCs from divergent strains, including LASV (OW), MACV (NW), and MOPV (OW), and this strategy yielded one MAb, KL-AV-2A1, with strong cross-reactivity against both OW and NW viruses. Another MAb derived from this strategy, KL-AV-IG12, showed cross-reactivity between the OW virus GPCs tested and had limited reactivity to some NW viruses as well. The third isolated MAb, KL-AV-1H9, was specific to LASV. While these numbers are not representative of the numerous antibodies present in the serum after exposure to an antigen, it is noteworthy that the strongly cross-reactive MAb was isolated after vaccination with divergent strains. Four MAbs bound to linear/microconformational epitopes (active in a Western blot assay under reducing and denaturing conditions), while the other two MAbs did not recognize GPC in Western blot assays and therefore likely bind fully conformational epitopes. All six MAbs are specific to the GP2 subunit, which is perhaps unsurprising given the higher conservation of this subunit. In the past, antibodies have been generated against the GPC of arenaviruses that exhibit neutralizing activity. In one study, vaccinating mice with JUNV resulted in the isolation of seven monoclonal antibodies (MAbs) that bound specifically to the JUNV GPC glycoprotein, four of which were able to neutralize the virus. However, none of these antibodies cross-reacted to any other arenavirus (31). A large panel of human MAbs was recently isolated from human survivors of Lassa fever. Of a total of 113 MAbs, 29 were specific to GP1, 27 bound specifically the whole GPC but not the individual subunits, and 57 bound to GP2 (20). Some of the GP2-targeting MAbs from that study showed limited cross-reactivity against other arenavirus GPCs. Finally, in a paper from 1991, Ruo and colleagues were able to isolate GP2-specific monoclonal antibodies from mice immunized with OW arenavirus that cross-reacted with New World arenaviruses such as JUNV, MACV, Amapari virus, and others (32). The MAbs isolated in our study resemble the human MAbs derived from LASV infection survivors since we found some strong cross-reactive antibodies. Unfortunately, our MAbs did not show neutralizing activity *in vitro*, a characteristic that they also share with the 57 human anti-GP2 MAbs (20). In contrast, several anti-GP2 MAbs reported by Ruo and colleagues did neutralize LASV (32). It is possible that due to the low number of isolated MAbs, we missed potentially neutralizing or protective MAbs. Another possibility is that the reagents used for immunization, specifically the nonstabilized recombinant proteins, did not display neutralizing GP2 epitopes in the right conformation. A recent study that solved the structure of the LASV GPC suggests that most neutralizing epitopes are displayed only in the prefusion conformation (33). However, due to the inherent metastable nature of the arenavirus GPC, the nonstabilized, recombinant GPC ectodomains often separate into the GP1 and GP2 alone, with the released GP2 assuming the postfusion six-helix bundle conformation. Interestingly, this was the conformation preferentially bound by all six of our MAbs. Therefore, a sequential vaccination strategy with divergent arenavirus GPCs that have been stabilized via mutations might lead to the isolation of neutralizing and protective MAbs and perhaps to a broadly protective antiarenavirus vaccination strategy similar to the ones under development for influenza viruses (30, 34).

## MATERIALS AND METHODS

### Cells and viruses.

BSC40 cells (ATCC CRL-2761) and Vero.E6 cells (ATCC CRL-1586) were grown in Dulbecco’s modified Eagle’s medium (DMEM; Life Technologies) which was supplemented with antibiotics (100 units/ml penicillin-100 µg/ml streptomycin [Pen-Strep]; Gibco), buffer solution (HEPES [1 M]; Gibco), and 10% fetal bovine serum (FBS; HyClone). Sf-9 cells were passaged in TNM-FH medium (Gemini Bioproducts) with antibiotics (Pen-Strep) and 10% FBS, while BTI-TN-5B1-4 cells were grown in serum-free SFX-insect medium (HyClone) supplemented with antibiotics (Pen-Strep). Replication-competent recombinant VSV (VSV-LASV) was provided by Heinrich Feldmann at Rocky Mountain Laboratories (RML), National Institute of Allergy and Infectious Diseases (NIAID), and has been described previously (17–19). Recombinant vaccinia viruses expressing the glycoproteins of various arenaviruses were obtained from the Biodefense and Emerging Infections Research Resources Repository (BEI Resources). These include recombinant vaccinia viruses that express LASV Josiah GPC (NR-15493), LCMV Armstrong 53b GPC (NR-15497), MACV Carvallo strain GPC (NR-15501), GOTV INH-95551 GPC (NR-15486), WWAV AV 9310135 GPC (NR-15508), and JUNV XJ13 GPC (NR-15691) (16).

### Propagation and titration of viruses.

Recombinant vaccinia viruses were grown and titrated in BSC40 cells (35). Confluent monolayers of these cells were infected with 50 µl of viral stock obtained from BEI Resources in 5 ml of minimal essential medium (MEM; Gibco) supplemented with antibiotics, HEPES, glutamine (l-glutamine [200 mM]; Gibco), and sodium bicarbonate (sodium bicarbonate 7.5% solution; Gibco) for an hour at 37°C. After an hour, 35 ml of MEM was added to obtain a larger viral stock. After 48 h, cells were lysed by three freeze-thaw cycles by placing the virus on a mixture of ethanol and dry ice for 30 s and then letting the virus sit at room temperature for 30 s. Cells were centrifuged at 4,000 rotations per minute (rpm) in a benchtop centrifuge (Beckman Coulter; Allegra X-15R, SX4750A rotor), the supernatant was collected, and smaller aliquots were frozen at −80°C. VSV-LASV was propagated in Vero.E6 cells. A confluent monolayer of cells was infected with 100 µl of VSV-LASV mixed with 5 ml of MEM for an hour, and then 35 ml of additional MEM was added. Cells were incubated at 37°C, and cytopathic effects were observed in the monolayer. After 48 h, all the supernatant was collected and frozen at −80°C. LASV, strain Nig08-A18 (GU481070.1), was grown in Vero.E6 cells and titrated in a BSL-4 setting as described by Oestereich et al. (36). Viral titers in the blood samples from mice were also titrated using this method.

### Plaque assays.

To titrate the recombinant vaccinia viruses, confluent monolayers of BSC40 cells were infected with different dilutions of the viral stock starting from 1:10 to 1:1,000,000 in phosphate-buffered saline (PBS) for an hour to allow the virus to attach to the cells. After an hour, cells were overlaid with MEM that contained 2% Oxoid agar (Oxoid purified agar; Thermo Scientific). Cells were incubated at 37°C for 2 days and then fixed. Cells were stained with crystal violet solution (Sigma-Aldrich), and plaques were counted. Titration of VSV-LASV was done in a similar manner using Vero.E6 cells.

### Generation of mammalian expression vectors encoding full-length arenavirus GPC.

Full-length sequences of three arenavirus glycoproteins were obtained from GenBank and include LASV strain Nig08-A19 (GenBank accession number GU481072.1), MACV strain Carvallo (GenBank accession number KM198592.1), and MOPV strain Mozambique (GenBank accession number DQ328874.1). These sequences were commercially synthesized (Invitrogen GeneArt Gene Synthesis). For each GPC, the sequence encoding the open reading frame was amplified and cloned into a pCAGGS mammalian expression vector. Each vector was Sanger sequenced (Macrogen), and DNA was purified using the PureLink HiPure Plasmid Midiprep kit (Invitrogen).

### Recombinant arenavirus GPCs.

The open reading frames encoding the ectodomain of nine arenavirus glycoproteins which include LASV Nig08-A19 (synthesized from GenBank accession number GU481072.1), MOPV (synthesized from GenBank accession number DQ328874.1), MACV Carvallo strain (synthesized from GenBank accession number KM198592.1), GOTV (synthesized from GenBank accession number AY129247.1), WWAV (synthesized from GenBank accession number NC_010700), PICV (synthesized from GenBank accession number K02734), PARV (amplified from ATCC VR-667), Tamiami virus (synthesized from GenBank accession number AF485263), and Tacaribe virus (synthesized from GenBank accession number M20304) were cloned into a baculovirus shuttle vector in frame with a C-terminal T4 foldon trimerization domain and a hexahistidine tag sequence (11). A conserved glycine residue upstream of the transmembrane domain at position 423 was used as the termination point. Baculoviruses were generated as described previously in Sf-9 cells, and glycoproteins were expressed in BTI-TN-5B1-4 cells and purified from cell supernatants according to a published protocol (11, 37). Recombinant LASV (Josiah) GP constructs used in ELISAs (Fig. 4B) were expressed in *Drosophila* S2 insect cells and purified by Strep-Tactin affinity chromatography and gel filtration as previously described (21, 23).

### Generation and screening of hybridomas.

Six- to 8-week-old female BALB/c mice (Jackson Laboratories) were vaccinated with 40 µg of pCAGGS vector encoding full-length GPC of either LASV Nig08-A19, MACV strain Carvallo, or MOPV. The DNA was administered via the intramuscular route, and an electrical stimulus was applied immediately after injection (TriGrid delivery system; Ichor Medical Systems) (23). The vaccination was performed for a total of three times with intervals of 2 to 3 weeks. One group of mice was vaccinated with only the plasmid coding for the LASV GPC to generate hybridomas specific for this virus. To induce a cross-reactive response, mice were sequentially vaccinated with vectors that encoded LASV GPC, MACV GPC, and finally MOPV GPC. Three days prior to the fusion, mice were boosted with 100 µg of recombinant LASV GPC adjuvanted with 10 µg of poly(I:C). On the day of the fusion, mice were sacrificed and the spleens were removed. The hybridoma fusions were performed according to a previously published detailed protocol (13, 38). Briefly, the harvested splenocytes were fused with SP2/0 myeloma cells using polyethylene glycol (Sigma-Aldrich), and cells were grown on semisolid Clonacell-HY Medium D (StemCell Technologies). Distinct colonies were picked and transferred to 96-well cell culture plates and grown in Medium E (StemCell Technologies) for 5 days. On the 5th day, an enzyme-linked immunosorbent assay (ELISA; procedure described below) was performed using 30 µl of hybridoma supernatant as primary antibody. Polyclonal serum collected from the immunized mice was used as a positive control at a dilution of 1:100. Mice that were vaccinated with DNA from divergent arenavirus GPCs were screened against all three GPs. Hybridomas that reacted positively on the ELISA were further isotyped using the Pierce rapid antibody isotyping kit (Life Technologies). IgG-secreting hybridomas were selected, passaged, and expanded to 300 ml of serum-free hybridoma medium (Hybridoma-SFM; Gibco). Antibodies were then purified using affinity chromatography columns packed with protein G Sepharose 4 Fast Flow (GE Healthcare) and used for further characterization. Expansion of hybridomas and purification of the hybridoma supernatants were performed as described in detail previously (14, 38).

### ELISA.

Plates (Immulon 4 HBX; Thermo Scientific) were coated with 2 µg/ml of the respective protein prepared in coating buffer (carbonate-bicarbonate buffer, pH 9.4) at 4°C overnight. The next morning, coating solution was removed and plates were blocked with 100 µl/well of 3% milk in PBS containing 0.1% Tween 20 (TPBS) for an hour at room temperature. Primary antibody solutions were prepared in TPBS containing 1% milk starting at a desired concentration, and 1:3 serial dilutions were prepared. Plates were incubated with primary antibody for an hour, after which plates were washed three times with 100 µl/well with plain TPBS. The secondary antibody used was a horseradish peroxidase (HRP)-labeled anti-mouse IgG (GE Healthcare) at a dilution of 1:3,000 prepared in 1% milk in TPBS. Plates were washed vigorously three times with TPBS and then developed using SigmaFast OPD (*o*-phenylenediamine dihydrochloride; Sigma-Aldrich). The OPD substrate was left on the plates for 10 min, after which the reaction was stopped with 50 µl/well of 3 M hydrochloric acid (HCl), and the plates were read at an optical density of 490 nm on a Synergy 4 (BioTek) plate reader. ELISAs with recombinant LASV GP constructs in Fig. 4B were performed using a modified method. Ninety-six-well half-area assay plates (Corning Incorporated) were coated with 50 µl at a final concentration of 0.5 µg per well in PBS. Plates were incubated overnight at 4°C and washed three times with TPBS. Plates were then blocked for 1 h with 125 µl of blocking buffer consisting of 3% bovine serum albumin (BSA)-PBS and then washed as described above. Each MAb was diluted to a final concentration of 5 µg/ml in blocking buffer and was added at a final volume of 50 µl/well. The plates were incubated for 1 h at 37°C and then washed as described above. Detection was performed with 50 µl/well of HRP-conjugated goat anti-human IgG antibody (Southern Biotech) diluted to 1:2,500 in blocking buffer. After a 1-h incubation, 50 µl/well of tetramethylbenzidine (TMB)-H_2_O_2_ substrate was added, and the plates were incubated for 5 min. The reaction was stopped by adding 50 µl/well of TMB stop solution and read at 450 nm.

### SDS-PAGE and Western blotting.

The samples were heated in 2× Laemmli buffer with 2% beta-mercaptoethanol (BME) at 100°C for 20 min and run on polyacrylamide gels (5% to 20% gradient; Bio-Rad). Gels were stained with SimplyBlue SafeStain (Invitrogen) for an hour and then destained in water for another hour. For Western blot assays, SDS-PAGE was performed with the sGPCe side by side with an irrelevant control protein (recombinant influenza A virus H7 protein). The gels were then transferred onto nitrocellulose membranes according to a previously described protocol (39). Each monoclonal antibody was used for primary staining at 30 µg/ml, and the secondary staining was performed using anti-mouse IgG (whole molecule)–alkaline phosphatase (AP) antibody produced in goat (Sigma-Aldrich) at a dilution of 1:1,000. The membrane was developed using an AP conjugate substrate kit, catalog no. 1706432 (Bio-Rad). To ensure that the proteins were transferred onto the nitrocellulose membrane, an anti-His monoclonal antibody (TaKaRa Bio Company) was used as a positive control, detecting a hexahistidine tag on the GPCs and the control protein.

### PRNA.

*In vitro* plaque reduction neutralization assays were performed using Vero.E6 cells according to an established protocol (13, 38). One hundred PFU of virus was incubated with dilutions of each antibody for 1 h at room temperature. Cells were washed with PBS, and the virus-antibody mixture was incubated on the cells for 1 h at 37°C. The mixture was then removed from the cells, and an agar overlay that contained serial dilutions of the antibodies was added to the cells. The cells were incubated in a cell culture incubator at 37°C for 48 h postinfection and stained with crystal violet (Sigma-Aldrich). The plaques were counted, and the data were analyzed with Prism 7 (GraphPad).

### *In vivo* studies in *Stat2*^*−/−*^ mice.

The *in vivo* studies were performed according to a similar design as described in detail previously (23). *Stat2*^−/−^ C57BL/6 mice were originally provided by Christian Schindler and were bred at the Icahn School of Medicine at Mount Sinai. Four groups consisting of two female and two male mice each, 6 to 8 weeks old, were infected with 1 PFU, 10 PFU, 100 PFU, and 1,000 PFU of VSV-LASV via the intraperitoneal route. Mice were monitored daily for survival and weight loss. Mice that lost 25% or more of their initial body weight were euthanized. The Reed and Muench method was used to obtain the mLD_50_. A prophylactic setting was used to assess whether an antibody is able to protect a mouse from infection or not. Each antibody group consisted of four mice, two males and two females. Mice were injected with 10 mg/kg of each antibody 2 h prior to infection with VSV-LASV via the intraperitoneal route (right flank). Mice were subsequently infected with 10 times the mLD_50_, and weight loss was monitored and recorded daily for 14 days. An irrelevant murine IgG that binds to hemagglutinin of influenza virus was used as a negative control. Survival curves and weight loss curves were created in Prism (GraphPad).

### *In vivo* studies with LASV strain Nig08-A18.

Chimeric mice were produced using female IFNAR^−/−^ mice (C57BL/6 background) that were irradiated and received 2 × 10^6^ bone marrow cells from coisogenic donor mice. The detailed protocol has been published before (40, 41). The mice were kept in groups of three to five mice per group in individually ventilated cages with water and food *ad libitum*. Eight weeks after the bone marrow transplantation, the engraftment was checked in peripheral blood by flow cytometry, and only mice with an engraftment above 85% donor cells were used in the experiments. Five mice per group were injected intraperitoneally with 15 mg/kg of the antibody KL-AV-1A2, KL-AV-1A12, KL-AV-1G12, or KL-AV-2A1 or an isotope control antibody. The antibodies were diluted in sterile PBS. Two hours after the antibody administration, the mice were infected intranasally with 1,000 focus-forming units (FFU) of LASV strain Nig08-A18 in 50 µl sterile PBS. Weight, rectal core body temperature, and symptoms were recorded daily for 3 weeks. Clinical score was calculated for consciousness, appearance, eyes, and respiration. Additional points were assigned for diarrhea (1 point) and for weight loss (1 point for weight loss up to 19.9% and 3 points for weight loss up to 20% to 24.9%). Fever and body temperature also led to extra points and a higher clinical score. Mice were sacrificed by isoflurane overdosing and cervical dislocation when criteria for euthanasia (weight loss of >80% of starting weight, symptom score of >10) were fulfilled or at the end of the experiment. Blood samples were collected periodically unless mice had a clinical score greater than 6 or had already succumbed to infection.

### ADCC reporter assay.

An ADCC reporter bioassay kit (Promega) was used to assess whether any of the MAbs elicit ADCC activity. The protocol has been described previously but was modified as needed (14, 23). Vero.E6 cells (50,000 per well) were added onto white round-bottom 96-well cell culture plates (Corning Costar) and infected with VSV-LASV at a multiplicity of infection (MOI) of 1.0. Virus was prepared in MEM, and this medium was removed from the cells after 16 h. Twofold serial dilutions of each antibody were added to the cells in duplicate starting from a concentration of 100 µg/ml. Seventy-five thousand effector cells were added to the cells with the antibody dilutions, and cells were incubated at 37°C for 6 h. Luciferase substrate was added, and luminescence was measured 2 to 5 min later using a Synergy Hybrid Reader (BioTek). Human monoclonal antibody CR9114 was used on influenza virus (A/duck/Czechoslovakia/1956 H4N6)-infected Vero.E6 cells as a positive control (42, 43).

### Immunofluorescence assays.

BSC40 cells were plated on 96-well cell culture plates and infected with each recombinant VACV in 1× MEM (Life Technologies) at an MOI of 1.0 overnight or for 16 h. The next morning, 150 µl of 3.7% paraformaldehyde was used to fix and inactivate VACV for 2 h. Cells were then incubated at room temperature with a blocking solution made of 3% milk in 1× PBS (Life Technologies) for 1 h. Each antibody was diluted to a concentration of 30 µg/ml in 1% milk in 1× PBS, and 100 µl of antibody solution was added to the cells for an hour. Cells were washed twice with PBS, and 100 µl of a 1:1,000 dilution of goat anti-mouse IgG heavy plus light chain (H+L)–Alexa Fluor 488 (Abcam) was added for 50 min. The secondary antibody was removed, and cells were kept in PBS to analyze immunofluorescence under the microscope (Olympus IX-70). The protocol was adapted from the work of Tan et al. (13). To test binding of antibodies to the wild-type LASV Nig08-A18 strain, Vero.E6 cells were grown on coverslips and were infected with the virus at an MOI of 0.1. Twenty-four hours postinfection, cells were counterstained with tetramethyl rhodamine isocyanate (TRITC)-labeled cell mask at a dilution of 1:1,000 for 10 min at room temperature. Cells were then fixed with 4% formaldehyde in PBS and permeabilized with 0.1% Triton X-100 in PBS. Cells were then stained with each antibody at 10 ng/µl for 60 min. Cells were washed carefully with PBS three times, after which cells were stained with a fluorescein isothiocyanate (FITC)-labeled anti-mouse secondary antibody and DAPI (4′,6-diamidino-2-phenylindole) at a dilution of 1:10,000 in PBS for 60 min. Cells were washed three times and mounted on glass slides with Prolong antifade mounting medium. Images of the stained cells were taken via confocal fluorescence microscopy.

### Conservation map of arenavirus glycoproteins and phylogenetic analysis.

Amino acid sequences from the indicated virus glycoproteins (or whole-genome sequences) were downloaded from GenBank with a preference for whole-genome sequences acquired from the field in a similar manner. These were then standardized by removal of all primer sequences and then (if applicable) translated using ExPASy (Swiss Institute of Bioinformatics), and amino acid sequences were extracted and labeled with short names as indicated below. A multiple sequence alignment (MSA) was generated using Clustal Omega, and the alignment file was analyzed using Jalview. Percent identity per position was then determined in Jalview as the percentage of utilized viral sequences that share the Lassa virus amino acid at each position. These percent identity values were then translated to heat map colors (blue is 100% shared; red is 0% shared) and visualized in Microsoft Excel. The map was then labeled and standardized so that each pixel corresponds to one amino acid in Adobe Illustrator CS6. The phylogenetic tree of the different GPCs was built using the neighbor-joining method based on amino acid sequences in Clustal Omega. The tree was then visualized and labeled in Figtree v1.4.1.3.

### Data availability.

The data that support the findings of this study are available from the corresponding author upon request.

## References

[1] Shao J, Liang Y, Ly H 2015 Human hemorrhagic fever causing arenaviruses: molecular mechanisms contributing to virus virulence and disease pathogenesis. Pathogens 4:283–306. doi:10.3390/pathogens4020283. 26011826 PMC4493475

[2] Yun NE, Walker DH 2012 Pathogenesis of Lassa fever. Viruses 4:2031–2048. doi:10.3390/v4102031. 23202452 PMC3497040

[3] Schmitz H, Köhler B, Laue T, Drosten C, Veldkamp PJ, Günther S, Emmerich P, Geisen HP, Fleischer K, Beersma MF, Hoerauf A 2002 Monitoring of clinical and laboratory data in two cases of imported Lassa fever. Microbes Infect 4:43–50. doi:10.1016/S1286-4579(01)01508-8. 11825774

[4] Patterson M, Grant A, Paessler S 2014 Epidemiology and pathogenesis of Bolivian hemorrhagic fever. Curr Opin Virol 5:82–90. doi:10.1016/j.coviro.2014.02.007. 24636947 PMC4028408

[5] Burri DJ, da Palma JR, Kunz S, Pasquato A 2012 Envelope glycoprotein of arenaviruses. Viruses 4:2162–2181. doi:10.3390/v4102162. 23202458 PMC3497046

[6] Lenz O, ter Meulen J, Klenk HD, Seidah NG, Garten W 2001 The Lassa virus glycoprotein precursor GP-C is proteolytically processed by subtilase SKI-1/S1P. Proc Natl Acad Sci U S A 98:12701–12705. doi:10.1073/pnas.221447598. 11606739 PMC60117

[7] Pasquato A, Rochat C, Burri DJ, Pasqual G, de la Torre JC, Kunz S 2012 Evaluation of the anti-arenaviral activity of the subtilisin kexin isozyme-1/site-1 protease inhibitor PF-429242. Virology 423:14–22. doi:10.1016/j.virol.2011.11.008. 22154237 PMC3285533

[8] Rojek JM, Pasqual G, Sanchez AB, Nguyen NT, de la Torre JC, Kunz S 2010 Targeting the proteolytic processing of the viral glycoprotein precursor is a promising novel antiviral strategy against arenaviruses. J Virol 84:573–584. doi:10.1128/JVI.01697-09. 19846507 PMC2798452

[9] Rojek JM, Sanchez AB, Nguyen NT, de la Torre JC, Kunz S 2008 Different mechanisms of cell entry by human-pathogenic Old World and New World arenaviruses. J Virol 82:7677–7687. doi:10.1128/JVI.00560-08. 18508885 PMC2493302

[10] Abraham J, Corbett KD, Farzan M, Choe H, Harrison SC 2010 Structural basis for receptor recognition by New World hemorrhagic fever arenaviruses. Nat Struct Mol Biol 17:438–444. doi:10.1038/nsmb.1772. 20208545 PMC2920743

[11] Krammer F, Margine I, Tan GS, Pica N, Krause JC, Palese P 2012 A carboxy-terminal trimerization domain stabilizes conformational epitopes on the stalk domain of soluble recombinant hemagglutinin substrates. PLoS One 7:e43603. doi:10.1371/journal.pone.0043603. 22928001 PMC3426533

[12] Wang TT, Tan GS, Hai R, Pica N, Petersen E, Moran TM, Palese P 2010 Broadly protective monoclonal antibodies against H3 influenza viruses following sequential immunization with different hemagglutinins. PLoS Pathog 6:e1000796. doi:10.1371/journal.ppat.1000796. 20195520 PMC2829068

[13] Tan GS, Lee PS, Hoffman RM, Mazel-Sanchez B, Krammer F, Leon PE, Ward AB, Wilson IA, Palese P 2014 Characterization of a broadly neutralizing monoclonal antibody that targets the fusion domain of group 2 influenza A virus hemagglutinin. J Virol 88:13580–13592. doi:10.1128/JVI.02289-14. 25210195 PMC4248980

[14] Wohlbold TJ, Podolsky KA, Chromikova V, Kirkpatrick E, Falconieri V, Meade P, Amanat F, Tan J, tenOever BR, Tan GS, Subramaniam S, Palese P, Krammer F 2017 Broadly protective murine monoclonal antibodies against influenza B virus target highly conserved neuraminidase epitopes. Nat Microbiol 2:1415–1424. doi:10.1038/s41564-017-0011-8. 28827718 PMC5819343

[15] Tan GS, Krammer F, Eggink D, Kongchanagul A, Moran TM, Palese P 2012 A pan-h1 anti-hemagglutinin monoclonal antibody with potent broad-spectrum efficacy in vivo. J Virol 86:6179–6188. doi:10.1128/JVI.00469-12. 22491456 PMC3372189

[16] Kotturi MF, Botten J, Sidney J, Bui HH, Giancola L, Maybeno M, Babin J, Oseroff C, Pasquetto V, Greenbaum JA, Peters B, Ting J, Do D, Vang L, Alexander J, Grey H, Buchmeier MJ, Sette A 2009 A multivalent and cross-protective vaccine strategy against arenaviruses associated with human disease. PLoS Pathog 5:e1000695. doi:10.1371/journal.ppat.1000695. 20019801 PMC2787016

[17] Marzi A, Feldmann F, Geisbert TW, Feldmann H, Safronetz D 2015 Vesicular stomatitis virus-based vaccines against Lassa and Ebola viruses. Emerg Infect Dis 21:305–307. doi:10.3201/eid2102.141649. 25625358 PMC4313664

[18] Safronetz D, Mire C, Rosenke K, Feldmann F, Haddock E, Geisbert T, Feldmann H 2015 A recombinant vesicular stomatitis virus-based Lassa fever vaccine protects guinea pigs and macaques against challenge with geographically and genetically distinct Lassa viruses. PLoS Negl Trop Dis 9:e0003736. doi:10.1371/journal.pntd.0003736. 25884628 PMC4401668

[19] Garbutt M, Liebscher R, Wahl-Jensen V, Jones S, Möller P, Wagner R, Volchkov V, Klenk HD, Feldmann H, Ströher U 2004 Properties of replication-competent vesicular stomatitis virus vectors expressing glycoproteins of filoviruses and arenaviruses. J Virol 78:5458–5465. doi:10.1128/JVI.78.10.5458-5465.2004. 15113924 PMC400370

[20] Robinson JE, Hastie KM, Cross RW, Yenni RE, Elliott DH, Rouelle JA, Kannadka CB, Smira AA, Garry CE, Bradley BT, Yu H, Shaffer JG, Boisen ML, Hartnett JN, Zandonatti MA, Rowland MM, Heinrich ML, Martínez-Sobrido L, Cheng B, de la Torre JC, Andersen KG, Goba A, Momoh M, Fullah M, Gbakie M, Kanneh L, Koroma VJ, Fonnie R, Jalloh SC, Kargbo B, Vandi MA, Gbetuwa M, Ikponmwosa O, Asogun DA, Okokhere PO, Follarin OA, Schieffelin JS, Pitts KR, Geisbert JB, Kulakoski PC, Wilson RB, Happi CT, Sabeti PC, Gevao SM, Khan SH, Grant DS, Geisbert TW, Saphire EO, Branco LM, Garry RF 2016 Most neutralizing human monoclonal antibodies target novel epitopes requiring both Lassa virus glycoprotein subunits. Nat Commun 7:11544. doi:10.1038/ncomms11544. 27161536 PMC4866400

[21] Henry Dunand CJ, Leon PE, Huang M, Choi A, Chromikova V, Ho IY, Tan GS, Cruz J, Hirsh A, Zheng NY, Mullarkey CE, Ennis FA, Terajima M, Treanor JJ, Topham DJ, Subbarao K, Palese P, Krammer F, Wilson PC 2016 Both neutralizing and non-neutralizing human H7N9 influenza vaccine-induced monoclonal antibodies confer protection. Cell Host Microbe 19:800–813. doi:10.1016/j.chom.2016.05.014. 27281570 PMC4901526

[22] Tan GS, Leon PE, Albrecht RA, Margine I, Hirsh A, Bahl J, Krammer F 2016 Broadly reactive neutralizing and non-neutralizing antibodies directed against the H7 influenza virus hemagglutinin reveal divergent mechanisms of protection. PLoS Pathog 12:e1005578. doi:10.1371/journal.ppat.1005578. 27081859 PMC4833315

[23] Duehr J, Wohlbold TJ, Oestereich L, Chromikova V, Amanat F, Rajendran M, Gomez-Medina S, Mena I, tenOever BR, García-Sastre A, Basler CF, Munoz-Fontela C, Krammer F 2017 Novel cross-reactive monoclonal antibodies against Ebolavirus glycoproteins show protection in a murine challenge model. J Virol 91:e00652-17. doi:10.1128/JVI.00652-17. 28592526 PMC5533894

[24] He W, Chen CJ, Mullarkey CE, Hamilton JR, Wong CK, Leon PE, Uccellini MB, Chromikova V, Henry C, Hoffman KW, Lim JK, Wilson PC, Miller MS, Krammer F, Palese P, Tan GS 2017 Alveolar macrophages are critical for broadly reactive antibody-mediated protection against influenza A virus in mice. Nat Commun 8:846. doi:10.1038/s41467-017-00928-3. 29018261 PMC5635038

[25] Lennemann NJ, Herbert AS, Brouillette R, Rhein B, Bakken RA, Perschbacher KJ, Cooney AL, Miller-Hunt CL, Ten Eyck P, Biggins J, Olinger G, Dye JM, Maury W 2017 Vesicular stomatitis virus pseudotyped with Ebola virus glycoprotein serves as a protective, non-infectious vaccine against Ebola virus challenge in mice. J Virol 91:e00479-17. doi:10.1128/JVI.00479-17. PMC5553159 28615211

[26] Emonet SF, Seregin AV, Yun NE, Poussard AL, Walker AG, de la Torre JC, Paessler S 2011 Rescue from cloned cDNAs and in vivo characterization of recombinant pathogenic Romero and live-attenuated Candid #1 strains of Junin virus, the causative agent of Argentine hemorrhagic fever disease. J Virol 85:1473–1483. doi:10.1128/JVI.02102-10. 21123388 PMC3028888

[27] Pasquato A, Kunz S 2016 Novel drug discovery approaches for treating arenavirus infections. Expert Opin Drug Discov 11:383–393. doi:10.1517/17460441.2016.1153626. 26882218

[28] Zeitlin L, Geisbert JB, Deer DJ, Fenton KA, Bohorov O, Bohorova N, Goodman C, Kim D, Hiatt A, Pauly MH, Velasco J, Whaley KJ, Altmann F, Gruber C, Steinkellner H, Honko AN, Kuehne AI, Aman MJ, Sahandi S, Enterlein S, Zhan X, Enria D, Geisbert TW 2016 Monoclonal antibody therapy for Junin virus infection. Proc Natl Acad Sci U S A 113:4458–4463. doi:10.1073/pnas.1600996113. 27044104 PMC4843420

[29] Enria DA, Maiztegui JI 1994 Antiviral treatment of Argentine hemorrhagic fever. Antiviral Res 23:23–31. doi:10.1016/0166-3542(94)90030-2. 8141590

[30] Krammer F 2017 Strategies to induce broadly protective antibody responses to viral glycoproteins. Expert Rev Vaccines 16:503–513. doi:10.1080/14760584.2017.1299576. 28277797

[31] Sanchez A, Pifat DY, Kenyon RH, Peters CJ, McCormick JB, Kiley MP 1989 Junin virus monoclonal antibodies: characterization and cross-reactivity with other arenaviruses. J Gen Virol 70:1125–1132. doi:10.1099/0022-1317-70-5-1125. 2471803

[32] Ruo SL, Mitchell SW, Kiley MP, Roumillat LF, Fisher-Hoch SP, McCormick JB 1991 Antigenic relatedness between arenaviruses defined at the epitope level by monoclonal antibodies. J Gen Virol 72:549–555. doi:10.1099/0022-1317-72-3-549. 1706408

[33] Hastie KM, Zandonatti MA, Kleinfelter LM, Heinrich ML, Rowland MM, Chandran K, Branco LM, Robinson JE, Garry RF, Saphire EO 2017 Structural basis for antibody-mediated neutralization of Lassa virus. Science 356:923–928. doi:10.1126/science.aam7260. 28572385 PMC6007842

[34] Nachbagauer R, Krammer F 2017 Universal influenza virus vaccines and therapeutic antibodies. Clin Microbiol Infect 23:222–228. doi:10.1016/j.cmi.2017.02.009. 28216325 PMC5389886

[35] Trindade GS, Emerson GL, Sammons S, Frace M, Govil D, Fernandes Mota BE, Abrahão JS, de Assis FL, Olsen-Rasmussen M, Goldsmith CS, Li Y, Carroll D, Guimarães da Fonseca F, Kroon E, Damon IK 2016 Serro 2 virus highlights the fundamental genomic and biological features of a natural vaccinia virus infecting humans. Viruses 8:E328. doi:10.3390/v8120328. 27973399 PMC5192389

[36] Oestereich L, Rieger T, Neumann M, Bernreuther C, Lehmann M, Krasemann S, Wurr S, Emmerich P, de Lamballerie X, Ölschläger S, Günther S 2014 Evaluation of antiviral efficacy of ribavirin, arbidol, and T-705 (favipiravir) in a mouse model for Crimean-Congo hemorrhagic fever. PLoS Negl Trop Dis 8:e2804. doi:10.1371/journal.pntd.0002804. 24786461 PMC4006714

[37] Margine I, Palese P, Krammer F 2013 Expression of functional recombinant hemagglutinin and neuraminidase proteins from the novel H7N9 influenza virus using the baculovirus expression system. J Vis Exp (81):e51112. doi:10.3791/51112. PMC3970794 24300384

[38] Wohlbold TJ, Chromikova V, Tan GS, Meade P, Amanat F, Comella P, Hirsh A, Krammer F 2016 Hemagglutinin stalk- and neuraminidase-specific monoclonal antibodies protect against lethal H10N8 influenza virus infection in mice. J Virol 90:851–861. doi:10.1128/JVI.02275-15. 26512088 PMC4702667

[39] Wohlbold TJ, Nachbagauer R, Xu H, Tan GS, Hirsh A, Brokstad KA, Cox RJ, Palese P, Krammer F 2015 Vaccination with adjuvanted recombinant neuraminidase induces broad heterologous, but not heterosubtypic, cross-protection against influenza virus infection in mice. mBio 6:e02556-14. doi:10.1128/mBio.02556-14. 25759506 PMC4453582

[40] Pérez-Girón JV, Belicha-Villanueva A, Hassan E, Gómez-Medina S, Cruz JL, Lüdtke A, Ruibal P, Albrecht RA, García-Sastre A, Muñoz-Fontela C 2014 Mucosal polyinosinic-polycytidylic acid improves protection elicited by replicating influenza vaccines via enhanced dendritic cell function and T cell immunity. J Immunol 193:1324–1332. doi:10.4049/jimmunol.1400222. 24958904 PMC4111144

[41] Oestereich L, Lüdtke A, Ruibal P, Pallasch E, Kerber R, Rieger T, Wurr S, Bockholt S, Pérez-Girón JV, Krasemann S, Günther S, Muñoz-Fontela C 2016 Chimeric mice with competent hematopoietic immunity reproduce key features of severe Lassa fever. PLoS Pathog 12:e1005656. doi:10.1371/journal.ppat.1005656. 27191716 PMC4871546

[42] Dreyfus C, Laursen NS, Kwaks T, Zuijdgeest D, Khayat R, Ekiert DC, Lee JH, Metlagel Z, Bujny MV, Jongeneelen M, van der Vlugt R, Lamrani M, Korse HJ, Geelen E, Sahin Ö, Sieuwerts M, Brakenhoff JP, Vogels R, Li OT, Poon LL, Peiris M, Koudstaal W, Ward AB, Wilson IA, Goudsmit J, Friesen RH 2012 Highly conserved protective epitopes on influenza B viruses. Science 337:1343–1348. doi:10.1126/science.1222908. 22878502 PMC3538841

[43] Chromikova V, Zaragoza MA, Krammer F 2017 Generation of a serum free CHO DG44 cell line stably producing a broadly protective anti-influenza virus monoclonal antibody. PLoS One 12:e0183315. doi:10.1371/journal.pone.0183315. 28910287 PMC5598927

